# Characterization of a *Klebsiella pneumoniae* mutant strain wGF 1–2 with attenuated virulence, altered morphology, and reduced biofilm formation

**DOI:** 10.3389/fcimb.2026.1761564

**Published:** 2026-03-27

**Authors:** Zongmao Dai, Yue Hu, Anran Tai, Yabin Lu, Shixiong Hu, Juan Pan, Ying Xiao, Xuelian Ma, Qiang Fu, Hongqiong Zhao, Zhanqiang Su, Panpan Tong, Zhihui Hao, Gang Yao, Jinquan Wang

**Affiliations:** 1 College of Veterinary Medicine, Xinjiang Agricultural University, Urumqi, China; 2 Xinjiang Key Laboratory of New Drug Research and Development for Herbivores Animals, College of Veterinary Medicine, Xinjiang Agricultural University, Urumqi, China

**Keywords:** biofilm formation, *Klebsiella pneumoniae*, multi-omics, phage, virulence

## Abstract

**Introduction:**

The global rise of antimicrobial resistance has positioned multidrug-resistant *Klebsiella pneumoniae* as a critical health threat, necessitating alternative therapeutic strategies such as phage therapy. However, the long-term evolutionary consequences of phage-bacteria interactions remain poorly understood. This study characterizes a unique attenuated mutant, wGF 1-2, derived from a hypervirulent K. pneumoniae strain (GF) during phage isolation efforts.

**Methods:**

The wGF 1-2 mutant was serendipitously isolated during attempts to obtain lytic phages against the parental GF strain. We performed an integrated multi-omics and phenotypic characterization, including genomic sequencing, proteomic profiling, and transcriptomic analysis. Host-pathogen interactions were assessed using a murine infection model (evaluating survival and tissue colonization), and the impact on the gut microbiota was analyzed via metagenomics.

**Results:**

Compared to the parental strain, wGF 1-2 exhibited a significant reduction in biofilm formation and distinct morphological alterations. In a murine model, the mutant was avirulent, resulting in 100% survival even at a high challenge dose (10⁶ CFU), with minimal tissue colonization. Multi-omics analysis revealed extensive genomic structural variations (81 insertions and 64 deletions). Proteomic shifts included the downregulation of proteins involved in metal ion binding and metabolic pathways. Furthermore, infection with wGF 1-2 led to host inflammatory suppression and a restructuring of the gut microbiota characterized by an increase in beneficial *Bacteroidota*.

**Discussion:**

This study provides a comprehensive characterization of an attenuated *K. pneumoniae* mutant, wGF 1-2. The extensive genomic and phenotypic alterations observed highlight the significant evolutionary potential of bacterial pathogens during phage interactions. These findings underscore the necessity of thorough safety assessments, including evolutionary risk evaluations, for the future development of phage-based therapies.

## Introduction

*Klebsiella pneumoniae* (KP) is a Gram-negative bacterium, and an opportunistic pathogen that colonizes the respiratory or intestinal tracts of humans and animals (Paczosa and Mecsas, 2016). It has the potential to cause a range of infectious diseases including septicemia, pneumonia, liver abscess, urinary tract infections, arthritis, and meningitis (Kohler et al., 2007; Schelenz et al., 2007; Paczosa and Mecsas, 2016), posing a significant threat to human health and livestock production (Chen et al., 2021). With the increasing prevalence of multidrug-resistant strains in clinical settings, KP has emerged as a significant cause of severe infections worldwide^8^, in particular, their carbapenem-resistant strains (CRKP) have been explicitly classified by the World Health Organization (WHO) as a Critical Priority Pathogen on the Priority Pathogens List due to their extensive antimicrobial resistance and the scarcity of effective therapeutic options (Jesudason, 2024). Phage therapy, leveraging the specificity of phages to lyse pathogens, has emerged as a potential alternative. In recent years, several reports have documented the application of phage therapy in treating infections associated with Kp (Gou et al., 2025). However, the rapid lytic action of virulent phages may trigger ​endotoxin release, increasing the risk of sepsis (LaVergne et al., 2018). Moreover, phage therapy could accelerate bacterial evolution through gene recombination mechanisms or drive bacteria to modify the CRISPR-Cas system to escape phage infection (Jiang et al., 2024). However, its application predominantly focuses on the immediate lytic action of virulent phages. Consequently, the long-term evolutionary consequences of phage-bacterial coexistence, particularly those mediated by temperate phages through lysogenic conversion, remain poorly understood. The bacterial phenotypic changes induced by lysogeny—especially increased virulence and enhanced biofilm formation—are of particular significance, as they constitute key determinants of pathogenicity (Govind et al., 2009; Guerra et al., 2022; Singh and Kulkarni, 2022; Vyas et al., 2022; Wang et al., 2023; Yin et al., 2024) and serve as reservoirs for antibiotic resistance genes (Xia et al., 2025).

Unexpectedly, during the isolation of lytic phages against a hypervirulent KP strain (GF), we observed results that differed from the expected outcome: instead of forming clear lytic plaques, the phage-host interaction yielded turbid plaques with central bacterial growth, a morphology consistent with temperate phage plaque formation (Stocker, 1960). We subsequently isolated a bacterial clone from the center of such a plaque, designated wGF 1-2, prompting a fundamental shift in our research focus from phage therapy to the comprehensive characterization of this putative lysogen. It is crucial to state that the primary aim of this study is not to definitively establish the precise molecular mechanisms linking a putative lysogenic event to phenotypic changes—a task that requires extensive downstream validation through genetic complementation and mutagenesis. Instead, we comprehensively compare it to its parental GF strain to define the consequential biological attributes—phenotypic, genomic, and host-interaction—arising from this atypical interaction.

Through this integrated approach—spanning phenotyping, Oxford Nanopore Technologies (ONT) sequencing for genomic structural variants, proteomics, murine infection models, and host transcriptomic and metagenomic analyses—based on comparative analysis with the parental GF strain, we demonstrate that wGF 1–2 is an attenuated mutant exhibiting genomic and proteomic alterations, reduced virulence in animal models, and beneficial modulation of gut microbiota. This work not only highlights the potential for phage-bacterial encounters to yield unexpected evolutionary outcomes but also provides a rigorously characterized candidate for future development, emphasizing the necessity of a multifaceted assessment of phage applications. This foundational work is also essential to robustly establish the mutant’s properties before embarking on future mechanistic investigations.

## 2 Materials and methods

### 2.1 Phage isolation and induction

Phage isolation and identification proceeded as follows. Sewage samples (10 L) were collected from a treatment plant in Urumqi, sealed, and stored at room temperature for 24 h; after sedimentation, the precipitate was discarded and the supernatant was retained. Bacterial cryopreserved stocks were removed from a -40°C freezer, thawed, and streaked onto solid medium, after which the plates were inverted and incubated at 37°C for 16–18 h. Single colonies were then inoculated into sterile broth and cultured in a shaker for 6–8 h; this activation step was repeated 2–3 times. One milliliter of log-phase bacterial culture was combined with 25 mL of the reserved sewage sample, mixed by inversion ten times, and incubated with shaking for 16–18 h. The mixture was centrifuged at 11000 g for 10 min, and the supernatant was filtered through a 0.22 μm membrane to yield the initial phage lysate. For purification, 10 μL of this lysate was mixed with 90 μL of sterile broth to create a 10–^1^ dilution, and serial ten-fold dilutions were prepared up to 10^-10^. For each dilution, 200 μL was mixed with 200 μL of log-phase bacterial broth and added to 5 mL of semi-solid agar at 55°C; after mixing, the overlay was poured onto solid medium, allowed to solidify for 30 min, and then incubated inverted at 37°C for 6–8 h until plaques appeared. A single plaque was picked into 1 mL of sterile broth, agitated for 1 h, centrifuged at 11000 g for 10 min, and the supernatant was filtered to obtain a phage eluate. This eluate was again serially diluted to 10^-10^, and the overlay procedure was repeated; the purification cycle was performed 5–6 times until uniform, isolated plaques were obtained. For spot testing, a log-phase bacterial culture was spread evenly onto solid medium with a swab and dried for 15 min; 10 μL of phage solution was spotted onto the lawn, and after 30 min the plates were inverted and incubated at 37°C for 6–8 h to observe lysis. For mitomycin induction, single bacterial colonies were inoculated into 1 mL of broth and shaken for 6–8 h; the culture was centrifuged at 11000 g for 5 min, the supernatant discarded, and the pellet resuspended in 3 mL of fresh broth for an additional 3 h of incubation. One-milliliter aliquots were distributed into tubes, and mitomycin C was added to final concentrations of 0, 0.5, and 1 μg/mL; after mixing, incubation continued for 12 h, after which phages were harvested by centrifugation, filtration, and purified as described above.

### 2.2 Transmission electron microscope

Samples were diluted to appropriate concentrations, depending on the sample type. Generally, at least 100 μL of liquid samples should be provided and the appropriate concentration should be prepared. Use a copper or nickel mesh coated with a supporting film. The diluted sample is dropped into small holes (also known as copper grid) in the copper grid. Phosphotungstic acid (PTA) and uranium acetate were commonly used as negative staining solutions. These dyes have a strong scattering effect on the electron beam and can show a distinct dark background under TEM. The stain was quickly dropped onto the sample on the loading net to ensure that the stain covered the sample uniformly. The dye solution will surround and penetrate into the interior of the sample, forming a high-density shell that wraps the sample through physical adsorption.

### 2.3 Bacterial string test

In the identification study of cKP and hvKP, bacterial string test is the traditional research method to identify them. Purified colonies were picked using an inoculating ring, and colonies with a draw length of ≥5 mm were considered positive and defined as hvKP. The bacterial colony in the plate was dipped with the bacterial inoculation loop, the viscous string was slowly pulled up, and the viscous string was measured.

### 2.4 Gram staining

Drop a drop of purified water into the slide, dip appropriate amount of bacteria in the water with an inoculation ring, and then place the water above the alcohol lamp to dry. After the bacterial sample was fixed, a drop of crystal violet solution was added and left for one minute. After washing with water, add a drop of iodine solution and leave for one minute. After washing with water, a drop of decolorization solution was added and left for 30s. After washing with water, add a drop of safranin staining solution and let it stand for 1 minute. After washing with water, the specimens were observed under a microscope.

### 2.5 Bacterial biofilm assays

Then 200 μL bacterial suspension at stationary phase was added to 96-well plate and cultured at 37°C for 24 hours. BHI liquid medium was used as Control (Control). After washing with PBS, 200 μL of 0.5% crystal violet was added and stained for 30 min. The solution was discarded and 200 μL glacial acetic acid was added, and the Optical density (OD) was measured at 550 nm. The ability of biofilm formation was determined according to the following criteria: OD≤ODc was no biofilm formation ability; ODc < OD ≤ 2ODc was considered as weak film formation ability. 2ODc < OD ≤ 4ODc indicated medium membrane formation ability. OD > 4ODc means strong film forming ability (ODc is the average value of Control).

### 2.6 Acute toxicity test in mice

The experimental mice consisted of 121 KM mice with SPF level, obtained from Hunan Slaike Jingda Laboratory Animal Co., LTD. The experimental animal license and quality certificate number was SCXK (Xiang) 2021-0002. Experimental groups (Paczosa and Mecsas, 2016): NC group (n = 11) (Kohler et al., 2007); GF group (10^6^, 10^5^, 10^4^, 10^3^ and 10^2^ groups, with 11 mice in each group) (Schelenz et al., 2007); wGF 1–2 groups (10^6^, 10^5^, 10^4^, 10^3^ and 10^2^ groups, with 11 mice in each group). Each mouse in the control group was intraperitoneally injected with 200μl sodium chloride physiological solution, while each mouse in the experimental group was injected with 200μl of the corresponding concentration of bacterial solution. The total observation time was 48 hours. The death of mice was counted and recorded during the observation. After the observation, the fresh feces of the mice were collected for metagenomic sequencing, and the blood of the mice was collected for routine blood test. The mice were then sacrificed and dissected, and the organs were fixed in 4% paraformaldehyde for subsequent pathological detection, and some fresh livers were collected for subsequent transcriptome sequencing. Animal euthanasia in this study was performed using cervical dislocation, a method approved by the American Veterinary Medical Association (AVMA) Guidelines for Euthanasia. Following the 48-hour *in vivo* observation, we randomly selected 6 mice from each group (initial n=11 per group) to perform subsequent histopathological analysis, metagenomic/transcriptomic sequencing.

### 2.7 Histopathological sections and HE staining

The tissues were fixed, dehydrated and embedded to obtain complete tissue wax blocks. The slices were then cut into uniform sections in a microtome. Subsequently, dewaxing was performed. Finally, staining was performed. Paraffin sections were first stained with hematoxylin solution for 1 min, then rinsed with tap water, followed by differentiation with 1% alcohol hydrochloride for a few seconds and rinsed again. Subsequently, a bluing step was performed for 1 min using 1% aqueous ammonia solution, followed by rinsing, placement into eosin staining solution for short staining, and final thorough rinsing with clean water. Sections were subjected to 75% and 85% ethanol for 2 min each, absolute ethanol for 10 min, absolute ethanol again for 5 min, and finally transparent with xylene for 5 min. The sections were removed in xylene and sealed with neutral gum to ensure the integrity and long preservation of the sections. After the above steps, under the microscope, you will see that the nucleus appears dark blue, while the cytoplasm appears bright red, which is the typical result of HE staining experiments.

### 2.8 Bacterial proteomics sequencing

Bacterial proteomics sequencing analysis was used to analyze the differences in the content and function of different bacterial proteins. Different types of bacteria were collected and rapidly frozen in liquid nitrogen. The samples were transported on dry ice to Shanghai Zhongke New Life Biotechnology Co., Ltd. for bacterial proteomics sequencing.

### 2.9 Whole genome sequencing of phage and bacteria

Whole genome analysis was used to analyze the specific functional mechanisms of phages and bacteria. Phage and bacterial genomes were collected and rapidly frozen in liquid nitrogen. The samples were transported on dry ice to BENAGEN Technologies Co., Ltd. for whole-genome sequencing.

### 2.10 Oxford nanopore technologies sequencing

Genome assembly and structural variations (such as deletions, insertions, inversions and translocations) between GF and wGF 1-2/wGF 2–18 were analyzed using ONT sequencing performed by BENAGEN Technologies Co., Ltd.

### 2.11 Transcriptome and metagenomics analysis

To analyze the molecular mechanism of mouse injury caused by different Kp using transcriptome and metagenome. The liver, spleen and feces of the control group and the treatment group were collected and quickly frozen in liquid nitrogen. The samples were transported on dry ice to BENAGEN Technologies Co., Ltd. for transcriptome and metagenomics sequencing.

### 2.12 AI-Assisted language editing

During the preparation of this manuscript, the authors employed DeepSeek-R1 (DeepSeek, Hangzhou, China; https://www.deepseek.com), an AI-driven language processing platform, to enhance the linguistic clarity, grammatical accuracy, and academic tone of the text. All AI-generated suggestions were rigorously reviewed, modified as necessary, and ultimately approved by the authors. DeepSeek-R1 was not involved in the design of experiments, data interpretation, or formulation of scientific conclusions.

### 2.13 Statistical information

All data are expressed as the mean ± SD of triplicate experiments. The results were analyzed by unpaired, two-tailed Student t-test using GraphPad Prism 9 (GraphPad software, Inc., USA). The asterisks represent levels of significance. *P* < 0.05 (*) were considered statistically significant, and *P* < 0.01, *P* < 0.001 were marked as (**) and (***), respectively.

## 3 Results

### 3.1 Phenotypic characterization of the isolated bacteria

During the initial isolation of Kp phage, we obtained both the enriched supernatant of the original phage pL and its purified supernatant. Titration of the original supernatant against the hypervirulent Kp (hvKP) strain GF revealed plaque morphologies typical of lysogeny. Ultimately, we isolated three phages (pL, p0.5-6, and p1-8) and two lysogenic strains (wGF 1–2 and wGF 2-18). The complete separation procedure is detailed in **Supplementary Figure 1**.

**Figure 1:**
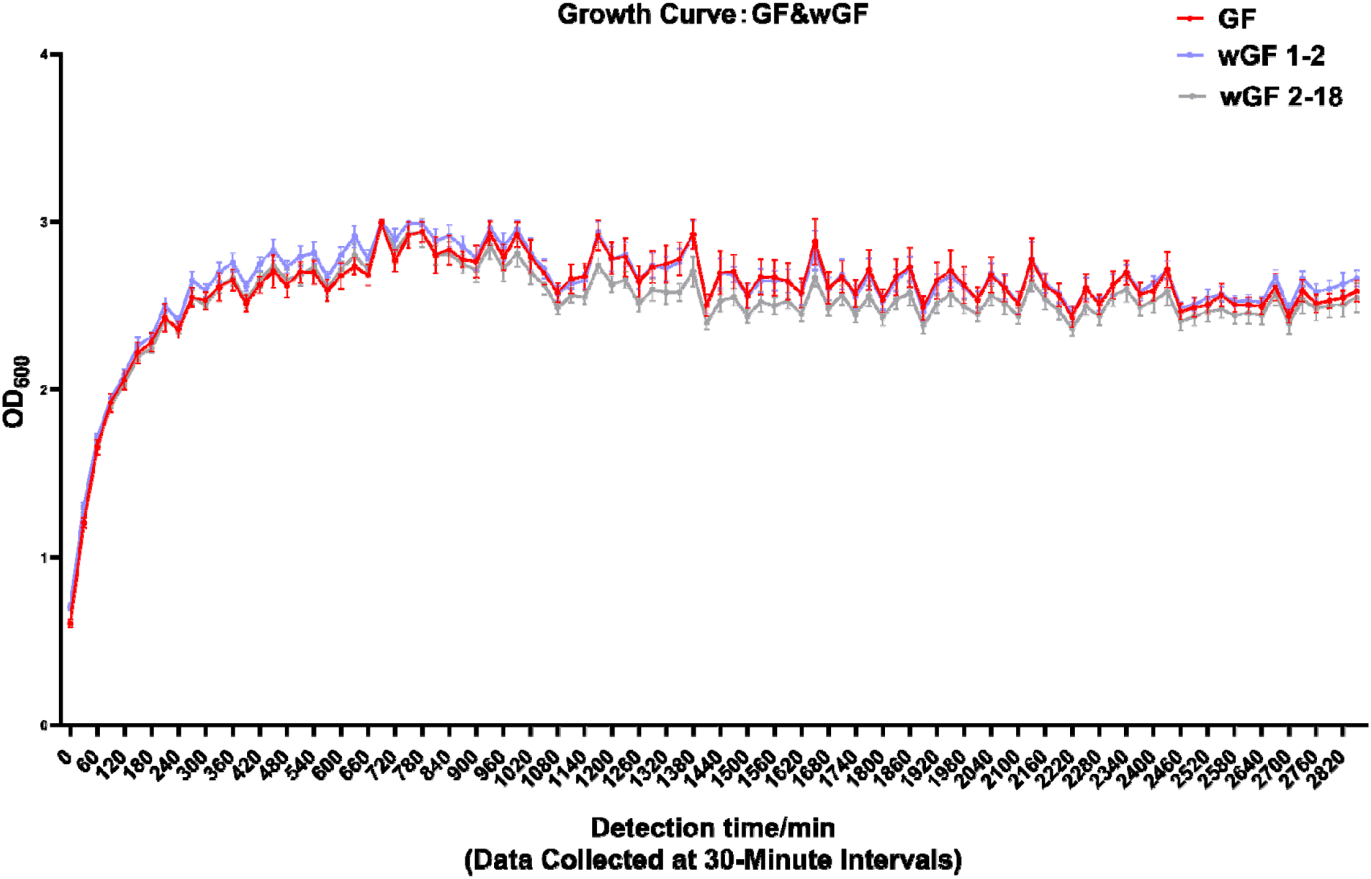
Bacterial growth curve. Correction was performed using BHI broth, and subsequent samples were tested at 30-minute intervals. Error bars represent mean ± SD. Data are representative of 12 biological replicates (n=3) with 3 technical replicates each.

**Figure 2:**
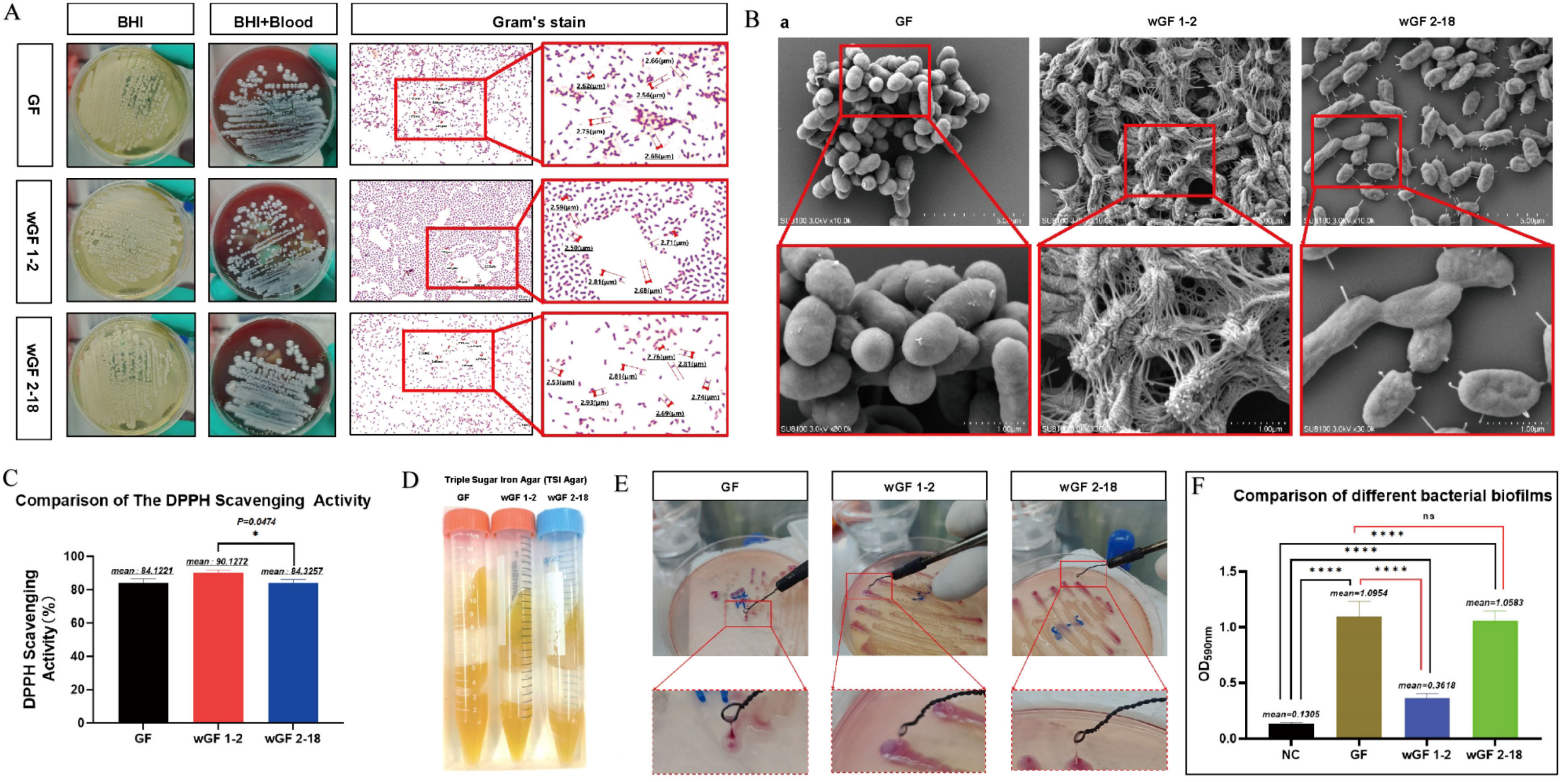
Basic biological characteristics of bacteria. **(A)** Bacterial growth morphology was observed in BHI solid medium and BHI-defibrinated sheep blood medium. Gram’s stain of bacteria. **(B)** Scanning electron microscopy of bacteria. **(C)** The detection of DPPH (2,2-Diphenyl-1-picrylhydrazyl) scavenging activity. **(D)** TSI agar. E Bacterial string test. F. The detection of bacterial biofilm. Error bars represent mean ± SD. Data are representative of 12 biological replicates (n=3) with 3 technical replicates each. * indicates P < 0.05, and **** indicates P < 0.0001.

First, by conducting basic biological characteristic analyses, investigate whether bacteria exhibit distinct phenotypes. As shown in **Figure 1**, the three strains exhibited similar growth patterns, all reaching their peak at approximately 600 minutes. On BHI agar, they formed colonies that were uniformly milky white and circular. On BHI-defibrinated sheep blood agar, the colonies were also consistent, appearing as grayish-white and circular without hemolytic rings. Gram staining revealed that all three strains were Gram-negative bacilli (**Figure 2A**). As depicted in **Figures 2B**, compared with GF, the two lysogenic strains exhibited morphological changes: wGF 1–2 showed significant cell wall shrinkage and dense filamentous connections between cells, while wGF 2–18 became flattened with a few protrusions on the cell wall. The DPPH (2,2-Diphenyl-1-picrylhydrazyl) scavenging ability of GF and wGF 2–18 was weaker than that of wGF 1-2; however, only wGF 2–18 showed statistically significant differences compared to wGF 1-2 (**Figure 2C**). The results of the triple sugar iron agar culture demonstrated that all three strains exhibited similar characteristics, with the medium turning yellow and showing stratification (**Figure 2D**). Finally, both the string test and biofilm synthesis assay indicated that wGF 1–2 had weaker performance compared to GF and wGF 2-18, with no significant difference observed between GF and wGF 2-18 (**Figures 2E, F**). The results of biochemical identification test (**Supplementary Table 1**) and antimicrobial susceptibility test (**Supplementary Table 2**) of the three strains showed consistent results.

### 3.2 Genomic and proteomic analyses reveal molecular characteristics of wGF 1-2

Basic biological characteristic analyses of the parent strain GF and two lysogenic strains (wGF 1–2 and wGF 2-18) revealed that wGF 1–2 exhibited the most obvious phenotypic variations compared to GF, including distinct cell wall morphology, altered antioxidant capacity, and reduced biofilm formation and string test performance.

**Figure 3:**
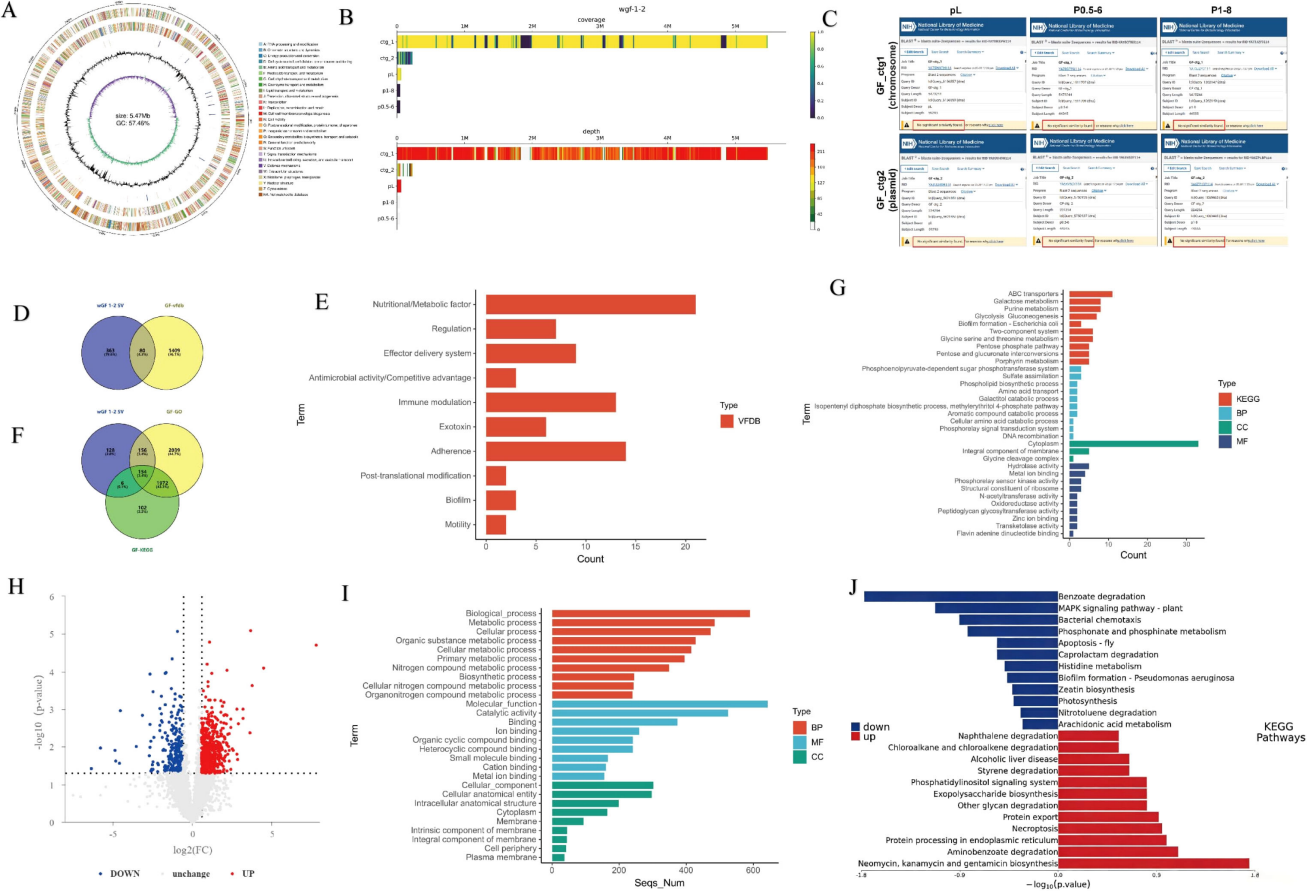
Multi-omics analysis of bacteria. **(A)** Map of the whole genome circle of GF. **(B)** Distribution of wGF 1–2 structural variants. **(C)** Alignment of phage and GF whole genome sequences. **(D)** Venn diagram of intersection of wGF 1–2 SV genes and GF-VFDB (Virulence factor database) genes. **(E)** VFDB annotation of wGF 1–2 SV genes. **(F)** Venn diagram of intersection of wGF 1–2 SV genes and GF-GO&KEGG genes. **(G)** GO&KEGG annotation of wGF 1–2 SV genes. **(H)** Proteomic volcano map of differential proteins. **(I)** Proteomics -GO enrichment. **(J)** KEGG enrichment of proteomics (wGF 1–2 vs GF). Error bars represent mean ± SD. Data are representative of 3 biological replicates (n=3) with 3 technical replicates each.Classification of gene functions: BP (Biological Process), MF (Molecular Function), CC (Cellular Component).

Due to its most prominent phenotypic variations, wGF 1–2 was prioritized for further investigation. To characterize structural variations in the wGF 1–2 genome, we performed ONT sequencing using the GF genome as a reference. Whole-genome sequencing of GF produced an assembly of 5.697 Mbp, consisting of a 5,473,244 bp chromosome and a 224,254 bp plasmid (**Figure 3A**). The strain was typed as ST23 by MLST, and annotation identified 16 prophages distributed across both the chromosome and the plasmid. ONT sequencing of wGF 1–2 revealed 81 insertions (INS), 64 deletions (DEL), 2 inversions (INV), 1 duplication (DUP), and 4 translocations (TRA) relative to the GF reference (**Figure 3B**). Mapping analysis showed that 5.85% of wGF 1–2 reads aligned to phage pL, while no significant alignment was detected for phages p0.5–6 or p1-8 (Minimap2, q-score ≥ 30; see **Supplementary Figure 3** for details). None of the three phages exhibited sequence homology with the GF chromosome or plasmid (**Figure 3C**).Using GF as the reference genome, functional annotation of structural variation (SV) genes in wGF 1–2 identified 80 virulence-related genes (4.3% of GF-VFDB (Virulence factor database), **Figure 3D**), which spanned categories including nutrient and metabolic regulation, effector delivery systems, antimicrobial activity, post-translational modification, adhesion, immune modulation, exotoxins, and biofilm formation (**Figure 3E**). A further 154 genes were annotated with GO/KEGG pathway associations (**Figure 3F**). KEGG enrichment analysis highlighted biofilm formation and two-component systems. Enriched biological processes included phospholipid biosynthesis, galactitol catabolism, and DNA recombination. Molecular functions were predominantly enriched in hydrolase activity, metal ion binding, peptidoglycan glycosyltransferase activity, and oxidoreductase activity, while cellular components were mainly associated with the cytoplasm, integral membrane components, and the glycine cleavage complex (**Figure 3G**). To further characterize molecular-level changes, we performed proteomic sequencing on GF and wGF 1-2. This analysis revealed 511 upregulated and 228 downregulated proteins (**Figure 3H**). GO analysis indicated that these differentially expressed proteins were primarily localized to the cytoplasm and cell membrane and were associated with metal ion binding (**Figure 3I**). KEGG analysis (**Figure 3J**) showed that upregulated proteins were enriched in the MAPK signaling pathway, bacterial chemotaxis, and biofilm formation. Downregulated proteins were enriched in pathways related to the degradation of biofilm components, such as other glycan degradation, naphthalene degradation, and chloroalkane/chloroalkene degradation.

### 3.3 Acute toxicity test in mice: GF vs wGF 1-2

**Figure 4:**
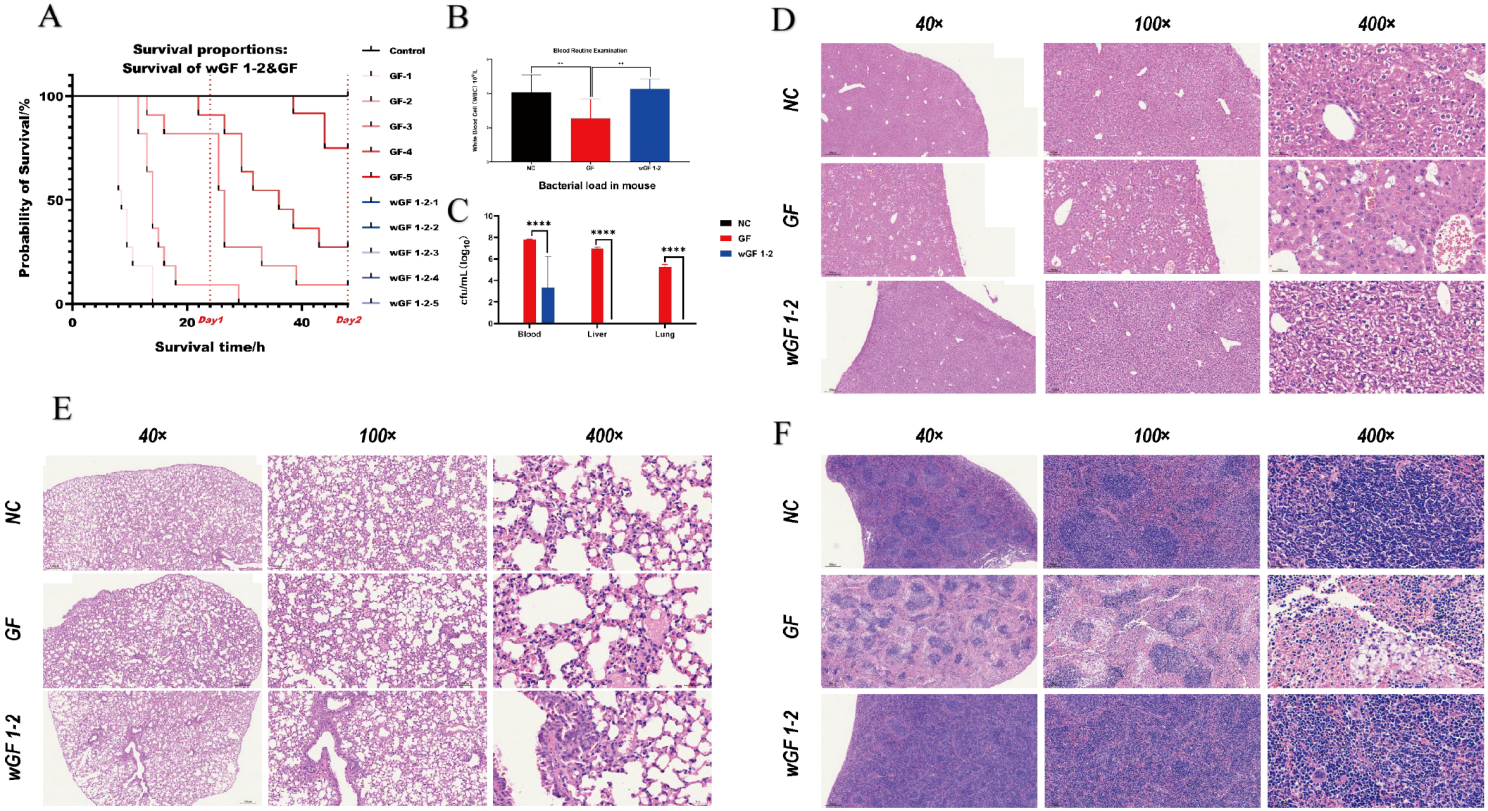
Acute toxicity test in mice: GF vs wGF 1-2. **(A)** Survival curve statistic. **(B)** Blood routine - white blood cell test. **(C)** Bacterial load statistics. Tissue sections **(D–F)** liver, lung and spleen. Error bars represent mean ± SD. The data are presented as 6 biological replicates per group, with 3 technical replicates performed for each biological replicate sample to ensure the stability of the detection results. ** indicates P < 0.01, and **** indicates P < 0.0001.

Based on these findings, we performed rigorous *in vivo* validation. In an acute toxicity assay, mice challenged with a high dose (10^^6^ CFU) of wGF 1–2 showed 100% survival, whereas the wild-type GF strain caused significant lethality (**Figure 4A**). The Reed-Muench method calculated an LD50 of 5 × 10^^5.50^ CFU/mL for GF, while wGF 1–2 was completely avirulent (0 CFU/mL) (**Supplementary Table 3**). Hematological analysis revealed that GF infection induced leukopenia (WBC: 2.83 × 10^9/L vs. 4.07 × 10^9/L in controls, *P* < 0.05), but wGF 1–2 did not reduce white blood cell counts (4.26 × 10^9/L) (**Figure 4B**). wGF 1–2 also showed a markedly low bacterial load in the blood and failed to colonize key organs like the liver and lungs (**Figure 4C**). Histopathology indicated only minor inflammation in the liver, spleen, and lungs of wGF 1-2-infected mice, contrasting sharply with the extensive necrosis and leukocyte infiltration observed following GF infection (**Figures 4D–F**). These results confirm the complete attenuation of wGF 1–2 virulence and its loss of invasive and colonizing capacity at the whole-animal level.

### 3.4 Integrated analysis of liver-spleen transcriptomics and feces metagenomics reveals functional enrichment profiles (GF VS wGF 1-2)

**Figure 5:**
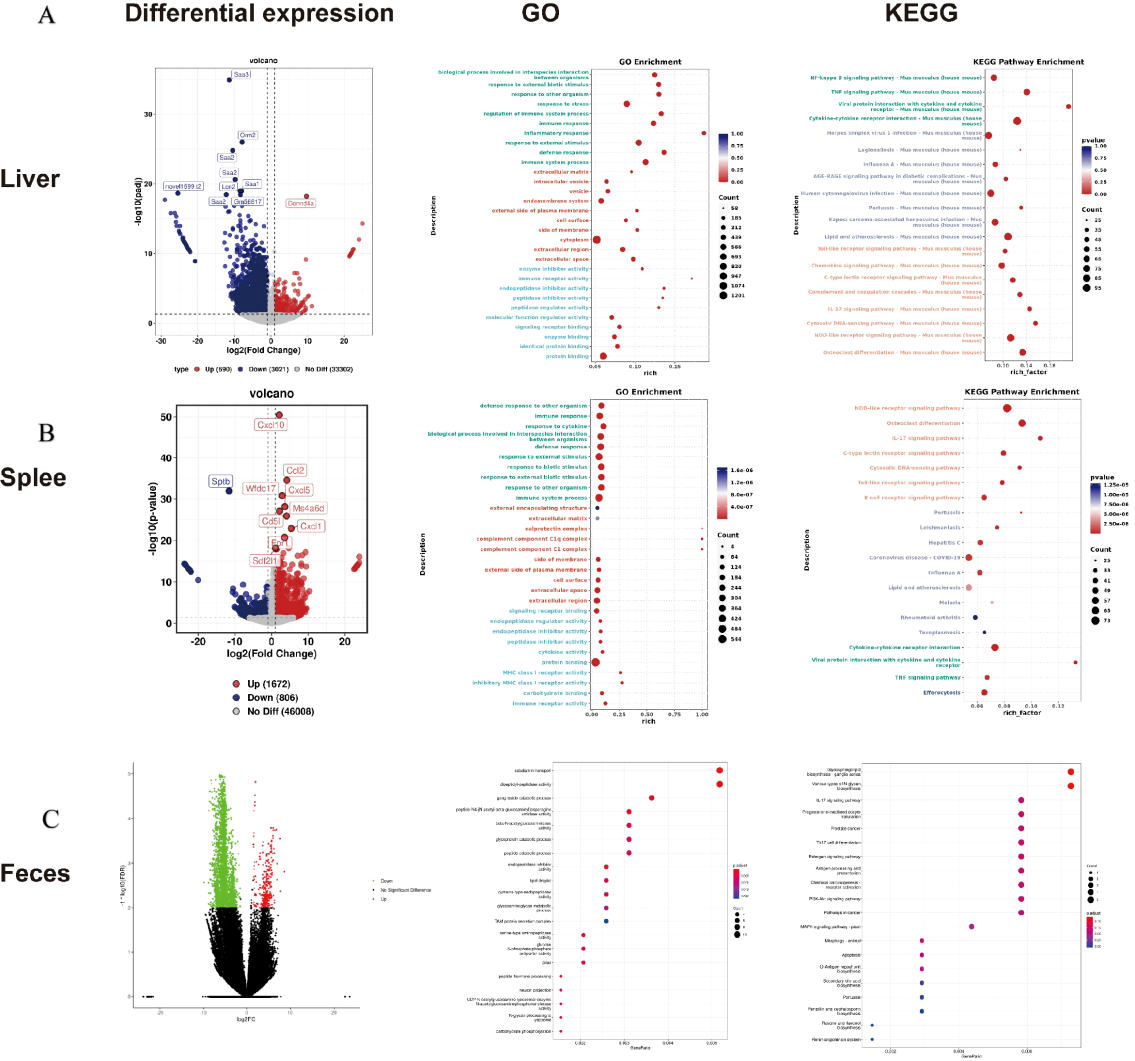
Functional enrichment analysis. Liver transcriptomics **(A)** Volcano plot of differentially expressed genes, GO and KEGG. Spleen transcriptomics **(B)** differential analysis volcano map, GO and KEGG. Feces - Metagenomics **(C)** Volcano plot of differentially expressed genes, GO and KEGG.Error bars represent mean ± SD. The data are presented as 6 biological replicates per group, with 3 technical replicates performed for each biological replicate sample to ensure the stability of the detection results.

Transcriptomic profiling of liver tissues identified 2,480 differentially expressed genes (629 upregulated and 1,851 downregulated), with KEGG enrichment analysis revealing suppression in key inflammatory pathways such as cytokine-cytokine receptor interaction, TNF-α signaling, Toll-like receptor, NOD-like receptor, and IL-17 signaling pathways (**Figure 5A**). A parallel pattern emerged from splenic transcriptomics, which identified 862 DEGs (644 upregulated and 218 downregulated) and whose functional enrichment confirmed analogous pathway alterations (**Figure 5B**). Metagenomic analysis identified 469 differentially expressed genes (361 upregulated and 108 downregulated), which were enriched in processes including glycosaminoglycan metabolism, lysosomal processing of N-glycans, glycosphingolipid biosynthesis, the IL-17 signaling pathway, the MAPK signaling pathway, Th17 cell differentiation, mitophagy, and apoptosis (**Figure 5C**).

### 3.5 The relative abundance of gut microbiota

**Figure 6:**
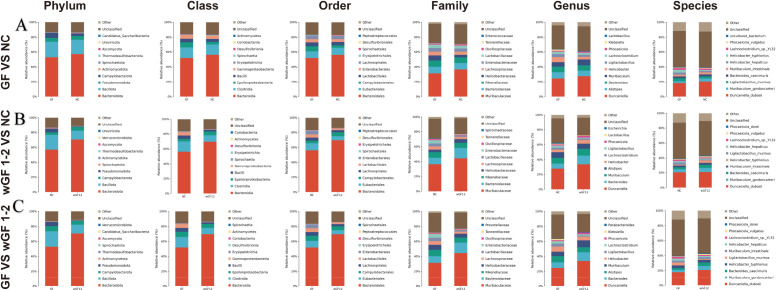
The relative abundance of gut microbiota (Phylum level→ species level). **(A)** GF vs NC. **(B)** wGF 1–2 vs NC. **(C)** GF vs wGF 1-2. Error bars represent mean ± SD. The data are presented as 6 biological replicates per group, with 3 technical replicates performed for each biological replicate sample to ensure the stability of the detection results. (NC, negative control).

Comparative analysis revealed distinct abundance gradients within the *Bacteroidota* lineage across experimental cohorts. The NC (control) group showed significantly higher relative abundances than the GF group across six consecutive taxonomic hierarchies (phylum to species), with *Bacteroidota* exhibiting elevated abundance that persisted through descendant taxa: *Bacteroidia* class, *Bacteroidales* order, and *Muribaculaceae* family. This vertical conservation extended to finer taxonomic levels, where the genus *Duncaniella* and its constituent species *Duncaniella_dubosii* were consistently enriched in the NC group (**Figure 6A**). In contrast, the wGF 1–2 cohort displayed markedly higher *Bacteroidota* abundance than the NC group at broader taxonomic levels (phylum to genus), including *Bacteroidia* class, *Bacteroidales* order, *Muribaculaceae* family, and *Duncaniella* genus. However, at the species level (*Duncaniella_dubosii*), no significant differences were observed between wGF 1–2 and NC groups, indicating divergence in hierarchical abundance patterns (**Figure 6B**). Compared to the GF group, wGF 1–2 maintained superior abundance across all taxonomic tiers of *Bacteroidota*, completing a conserved vertical gradient spanning phylum to genus (**Figure 6C**).

Compared with the NC group, the wGF 1–2 group exhibited altered intestinal microbiota affecting pathogenic colonization (1): decreased *γ-proteobacteria* and its lineages (*Enterobacterales*, *Enterobacteriaceae*, *Escherichia*) (2); increased *Campylobacterota* and its lineage (*Helicobacter_typhlonius*). In the GF group, both pathogen-related groups showed increased abundance.

## 4 Discussion

This study provides a comprehensive multi-omics characterization of the *Kp* mutant strain wGF 1-2, revealing alterations in its phenotypic properties, genomic architecture, and host interaction profiles. The mutant, serendipitously isolated during phage isolation attempts, demonstrates a unique combination of complete virulence attenuation alongside significant genomic reorganization, offering valuable insights into bacterial evolution under phage pressure.

wGF 1–2 displayed weak biofilm formation capacity and a negative string test result (**Figures 2E, F**), phenotypic traits closely associated with bacterial virulence. Previous studies have extensively confirmed that biofilm formation and mucoid phenotypes are key characteristics of hypervirulent *Kp*, promoting pathogenicity by enhancing bacterial colonization, immune evasion, and antibiotic tolerance (Pereira et al., 2021; Chang and Ong, 2022; Guerra et al., 2022). In this study, the biofilm deficiency of wGF 1–2 strongly correlated with its completely attenuated phenotype in animal models—even at high challenge doses (10^^6^ CFU), it demonstrated 100% survival rates in mice with minimal bacterial loads in tissues (**Figures 4A, C**).

Proteomic data further supported this connection, showing significant downregulation of pathways related to biofilm synthesis, such as “other glycan degradation” and “metal ion binding” (**Figures 3I, J**). Metal ions (e.g., iron) serve as crucial cofactors for biofilm matrix stability, and the downregulation of their binding proteins may directly lead to structural collapse of biofilms (O’May et al., 2009). These findings indicate that the virulence attenuation of wGF 1–2 may partially stem from its loss of biofilm formation capacity, consistent with the established consensus that biofilms positively correlate with virulence.

IL-17 upregulates inflammatory gene expression either by inducing *de novo* gene transcription or by stabilizing target mRNA transcripts (Amatya et al., 2017), thereby exerting a pro-inflammatory role in the host. Integrated liver/spleen transcriptomics and fecal metagenomics consistently revealed IL-17 signaling enrichment (**Figure 5**); IL-17 pathway activation was suppressed in wGF 1–2 vs. GF (consistent with IL-17’s pro-inflammatory role), corroborated by histopathology showing reduced inflammation and tissue damage in wGF 1-2 (**Figures 4D–F**); only the IL-17 pathway was consistently enriched across analyses, highlighting its core role in host anti-Kp response and therapeutic potential for hypervirulent Kp strains.

This study revealed significant morphological alterations in the attenuated mutant wGF 1-2, as visualized through scanning electron microscopy (**Figure 2B**). These structural changes were further supported by proteomic analyses, wherein GO enrichment indicated substantial impacts on cytoplasmic and cell membrane-associated components (**Figure 3I**). Concurrently, KEGG pathway analysis revealed upregulation in exogenous polysaccharide biosynthesis, potentially contributing to the observed intercellular filamentous connections (**Figure 3J**). A key finding was the markedly reduced colonization capability of wGF 1-2, evidenced by significantly lower bacterial loads in host tissues (**Figure 4C**). This impairment is likely linked to downregulated chemotaxis-related functions, aligning with established knowledge that compromised motility attenuates pathogenicity and tissue damage (Yoshiyama et al., 1999; Terry et al., 2005; Aihara et al., 2014). These morphological and metabolic shifts may reflect broader adaptive strategies under stress conditions. For instance, filamentation and cell wall remodeling are known bacterial responses to enhance survival under adverse conditions, such as antibiotic-induced DNA damage (Yu et al., 2023). In wGF 1-2, such alterations appear to modulate the exposure or conformation of pathogen-associated molecular patterns (PAMPs). Previous studies have demonstrated that changes in morphology—such as the transition of macrophages from a rounded to a flattened phenotype—enhance the capacity to recognize damage- and pathogen-associated molecular patterns (DAMPs/PAMPs) (Zhao et al., 2022), thereby reducing their recognition by host pattern recognition receptors (e.g., TLR4 and NOD2; **Figure 5**). This attenuated immune activation aligns with the observed dampening of pro-inflammatory pathways—such as IL-17 and TNF signaling—and corresponding reductions in tissue inflammation. In summary, these insights not only deepen our understanding of bacterial virulence evolution but also highlight potential targets for novel anti-virulence strategies.

Metagenomic profiling highlighted wGF1-2’s unique capacity to enrich *Bacteroidota* lineages, particularly *Duncaniella_dubosii*, in murine guts. *Bacteroidetes* are known to fortify gut barrier integrity via short-chain fatty acid (SCFA) production (Xiao et al., 2022) and suppress pro-inflammatory cytokines (Donaldson et al., 2018; Lloyd-Price et al., 2019; Coluzzi et al., 2024). Interestingly, functional enrichment analysis of bacterial proteomics, liver and spleen transcriptomics, and metagenomics all showed that wGF 1–2 is weaker than GF in inflammatory pathway activation, so whether *Bacteroidota* also play a role in this process needs to be further explored. However, therapeutic exploitation of this interaction requires caution, as *Bacteroidetes* enrichment may inadvertently promote pathogen replacement (e.g., *Clostridioides difficile*) (Round et al., 2011; Buffie et al., 2015). Similarly, while wGF 1–2 reduced potential pathogens such as γ-*proteobacteria* and spirochetes, the compensatory proliferation of *Campylobacterota* underscores the complexity of microbiota regulation (**Figure 6**).

Horizontal gene transfer (HGT) is a critical driver of bacterial evolution (Arnold et al., 2022; Goyal, 2022). As horizontally mobile elements, phages play a significant role in driving bacterial evolution through HGT (Brown-Jaque et al., 2015; Balcazar, 2018; Aljanazreh et al., 2023). Phages can be categorized into two types based on their life cycles: virulent phage and temperate phage (Clokie et al., 2011). Virulent phages complete their replication cycle by directly lysing the host, while temperate phages integrate into the host genome (prophage state) for latency and initiate lysis under environmental stress (Barksdale and Arden, 1974). Virulent phages are widely used therapeutically for their lytic efficacy, while temperate phages can stably alter bacterial phenotypes via lysogenic conversion (exemplified by our attenuated wGF 1–2 mutant). Multi-omics data link wGF 1-2’s genomic rearrangements and attenuation to phage pL lysogenization (supported by 5.85% read alignment, no homology in parental GF), correlating with reduced virulence, biofilm formation, and host inflammation, likely via PAMP alterations that reduce PRR recognition. This study lacks complementation assays to confirm a direct causal link between phage integration and phenotype. Future priorities are (1): CRISPR-based mapping of phage integration sites and their effects on adjacent gene expression (2); functional validation of key virulence attenuation pathways (3); safety and efficacy evaluation of wGF 1–2 as a live-attenuated vaccine against hypervirulent *Klebsiella pneumoniae*.

To better illustrate the overall experimental procedure, a schematic diagram was prepared and presented in **Supplementary Figure 2**.

## 5 Conclusions

In conclusion, wGF 1-2’s attenuation arises from interconnected genomic, proteomic, and host-level alterations, highlighting the complex outcomes of phage-bacterial interactions. These findings underscore the need for comprehensive safety assessments in phage-based applications while providing a valuable resource for studying bacterial virulence evolution.

## Data Availability

The data presented in the study are deposited in the iProX repository, accession number IPX001549700; the NCBI Sequence Read Archive (SRA) repository, accession numbers PRJNA1417531, PRJNA1416482, PRJNA1416566, PRJNA1416591; and the NCBI GenBank repository, accession numbers PX979744, PX979745, PX979746. All datasets are publicly available.
