# Supplementary material for: Characterization of a Klebsiella pneumoniae mutant strain wGF 1–2 with attenuated virulence, altered morphology, and reduced biofilm formation
Source: Front Cell Infect Microbiol. 2026 Mar 27;16:1761564. doi: 10.3389/fcimb.2026.1761564 (PMC13066129; doi:10.3389/fcimb.2026.1761564)
Supplement: Supplementary file 1 [file Table1.docx]

Supplementary Material

Isolation and identification of three strains of of *Klebsiella pneumonia* temperate bacteriophage

During the primary enrichment phase, co-cultivation of raw sewage with Kp strain L9 (L9) through a dual-phase purification strategy yielded two critical outputs: a first-generation crude lysate containing heterogeneous bacteriophage populations, and a *Myoviridae* bacteriophage pL isolated using the double-layer agar method on L9 lawns (Figure S1A, B). To delineate the host specificity spectrum, these preparations were challenged against hypervirulent *Klebsiella pneumoniae* (hvKP) strain GF (KL1 capsular serotype). Intriguingly, the lysate-GF interaction produced atypical plaques (Figure S1C) exhibiting bimodal characteristics: central microcolonies of lysogenized GF confirmed by prophage induction assays, surrounded by lytic clearance zones, indicative of concurrent lysogenic conversion and lytic activity. Subsequent mitomycin-C induction (Figure S1D,E) enabled isolation of two distinct *Podoviridae* morphotypes: p0.5-6 and p1-8 as characterized by transmission electron microscopy (Figure S1F, G).

Whole-genome sequencing (Illumina NovaSeq 6000, 2×150 bp) revealed pL's 44.0 kb dsDNA genome (53.91% GC content), p1-8's 44.5 kb dsDNA genome (53.81% GC content), and p0.5-6's 59.2 kb dsDNA genome (56.06% GC content) (Figure S1H-J), while comparative phylogenomic analysis using VICTOR (v1.0) with maximum-likelihood bootstrapping (1,000 replicates) positioned all three phages within the Kp Phage (Figure 1K).

Among these, the bacterial strains induced to express p0.5-6 were designated as Strain 5, while those induced to express p1-8 were designated as Strain 8. Subsequent isolation and purification of these two strains resulted in the successful acquisition of two lysogenic bacterial isolates, namely wGF 1-2 and wGF 2-18.


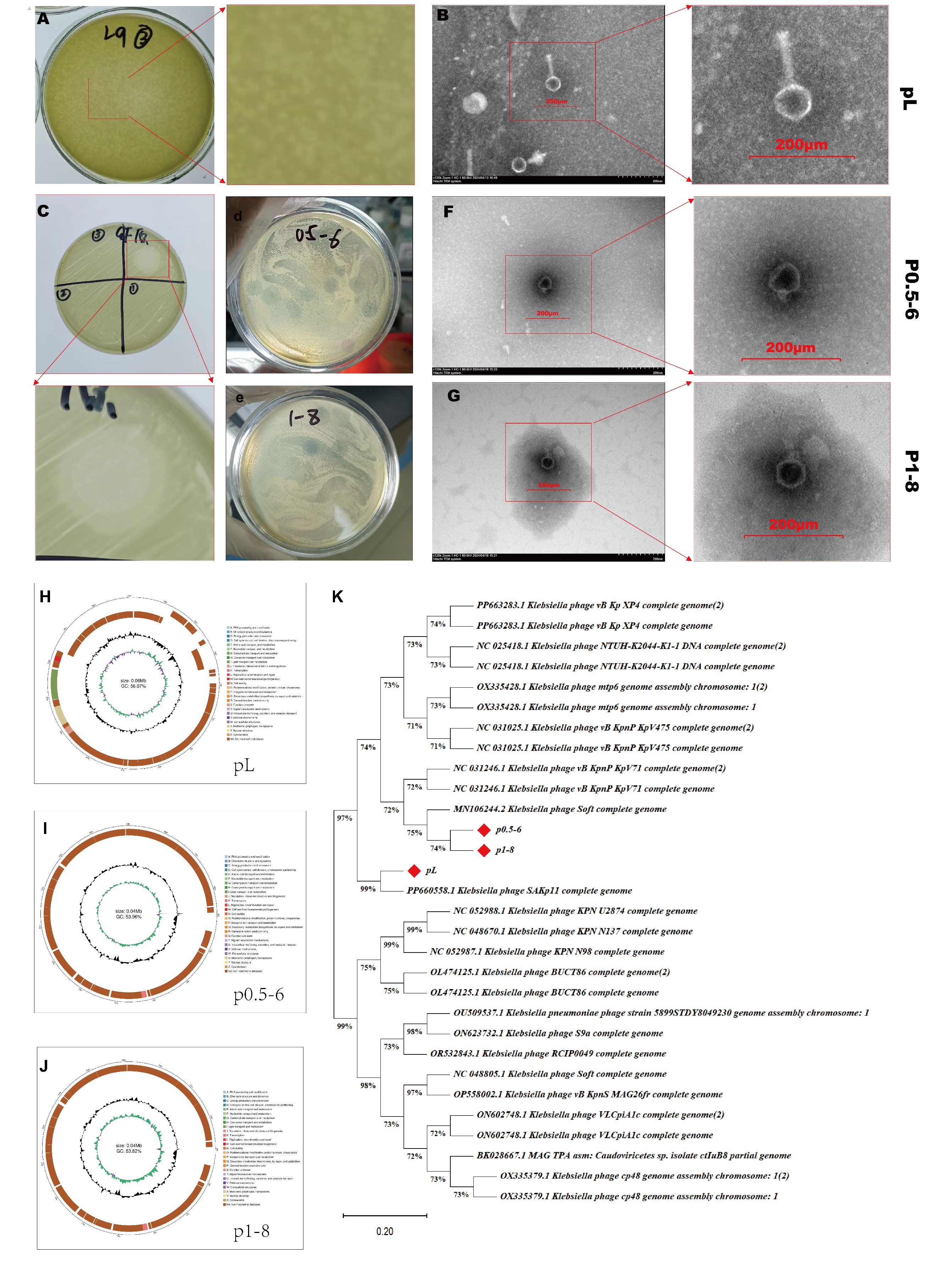


Supplementary Figure 1. Isolation and identification of three strains of of *Klebsiella pneumonia* temperate bacteriophage.

**Supplementary Table 1.** Biochemical Identification ( GF & wGF 1-2)

| Bacteria | Biochemical Reaction（-/+） | | | | | | | | | | | | | | | |
| --- | --- | --- | --- | --- | --- | --- | --- | --- | --- | --- | --- | --- | --- | --- | --- | --- |
|  | Semi-Solid Agar | Ornithine | Lysine | Hzs | Citrate | Urease | Peptone Water | MR | V-P | Phenylalanine | Mannitol | Inositol | Sorbitol | Meleediol | Ribitol | Raffinose |
| GF | + | - | + | - | + | + | - | - | + | - | + | + | + | + | + | + |
| 1-2 | + | - | + | - | + | + | - | - | + | - | + | + | + | + | + | + |
| 2-18 | + | - | + | - | + | + | - | - | + | - | + | + | + | + | + | + |

**Supplementary Table 2.** Statistics of antimicrobial susceptibility test results

| Type | Antibiotics | Bacteria | | |
| --- | --- | --- | --- | --- |
|  |  | GF | wGF 1-2 | wGF 2-18 |
| Penicillins | Penicillin | R | R | R |
| Macrolides | Clarithromycin | M | M | M |
| Glycopeptides | Vancomycin | R | R | R |
| Lincosamides | Clindamycin | R | R | R |
| Amphenicols | Chloramphenicol | S | S | S |
| Aminoglycosides | Kanamycin | S | S | S |
| Cephalosporins | Cefuroxime Sodium | S | S | S |
| Tetracyclines | Tetracycline | M | M | M |
| Polypeptides | Polymyxin B | S | S | S |
| Quinolones | Norfloxacin | S | S | S |
| Rifamycins | Rifampicin | R | R | R |
| Carbapenems | Imipenem | S | S | S |
| Sulfonamides | Co-trimoxazole | S | S | S |

S (Susceptible): Inhibition zone diameter ≥ clinical breakpoint, indicating effective antimicrobial activity

D (Dose-dependent Susceptibility): Requires dosage adjustment for clinical efficacy

M (Resistant by Modified Criteria): Resistant under specific testing conditions

| **Supplementary Table 3**.the statistical calculation of the LD50 of bacteria | | |
| --- | --- | --- |
| ID | Fatality Rare(%) | TCID_50_ |
| NC | 0.00 | 5×10^5.50^ LD_50_/ml |
| GF-1 | 1.00 |  |
| GF-2 | 1.00 |  |
| GF-3 | 0.91 |  |
| GF-4 | 0.73 |  |
| GF-5 | 0.27 |  |
| wGF 1-2-1 | 0.00 | 0LD_50_/ml |
| wGF 1-2-2 | 0.00 |  |
| wGF 1-2-3 | 0.00 |  |
| wGF 1-2-4 | 0.00 |  |
| wGF 1-2-5 | 0.00 |  |
