# Supplementary figures and images for: Characterization of a Klebsiella pneumoniae mutant strain wGF 1–2 with attenuated virulence, altered morphology, and reduced biofilm formation
Source: Front Cell Infect Microbiol. 2026 Mar 27;16:1761564. doi: 10.3389/fcimb.2026.1761564 (PMC13066129; doi:10.3389/fcimb.2026.1761564)

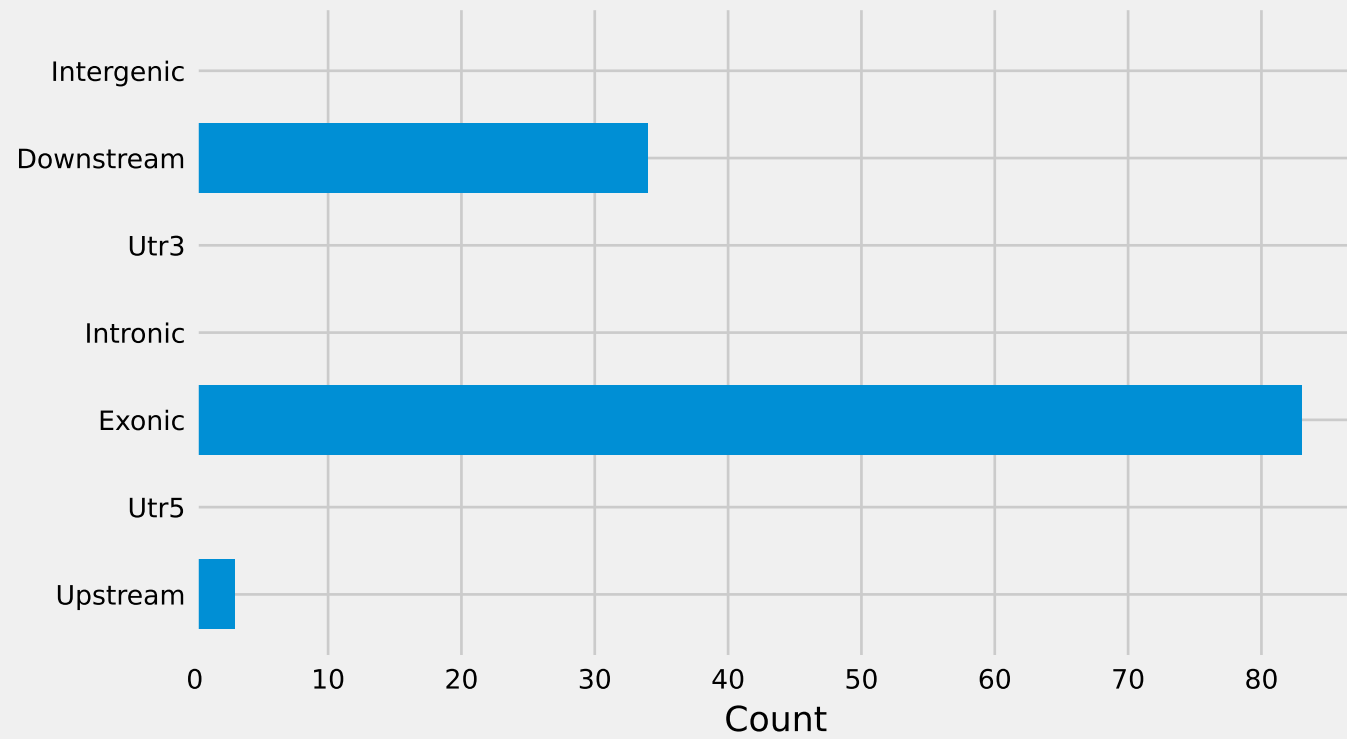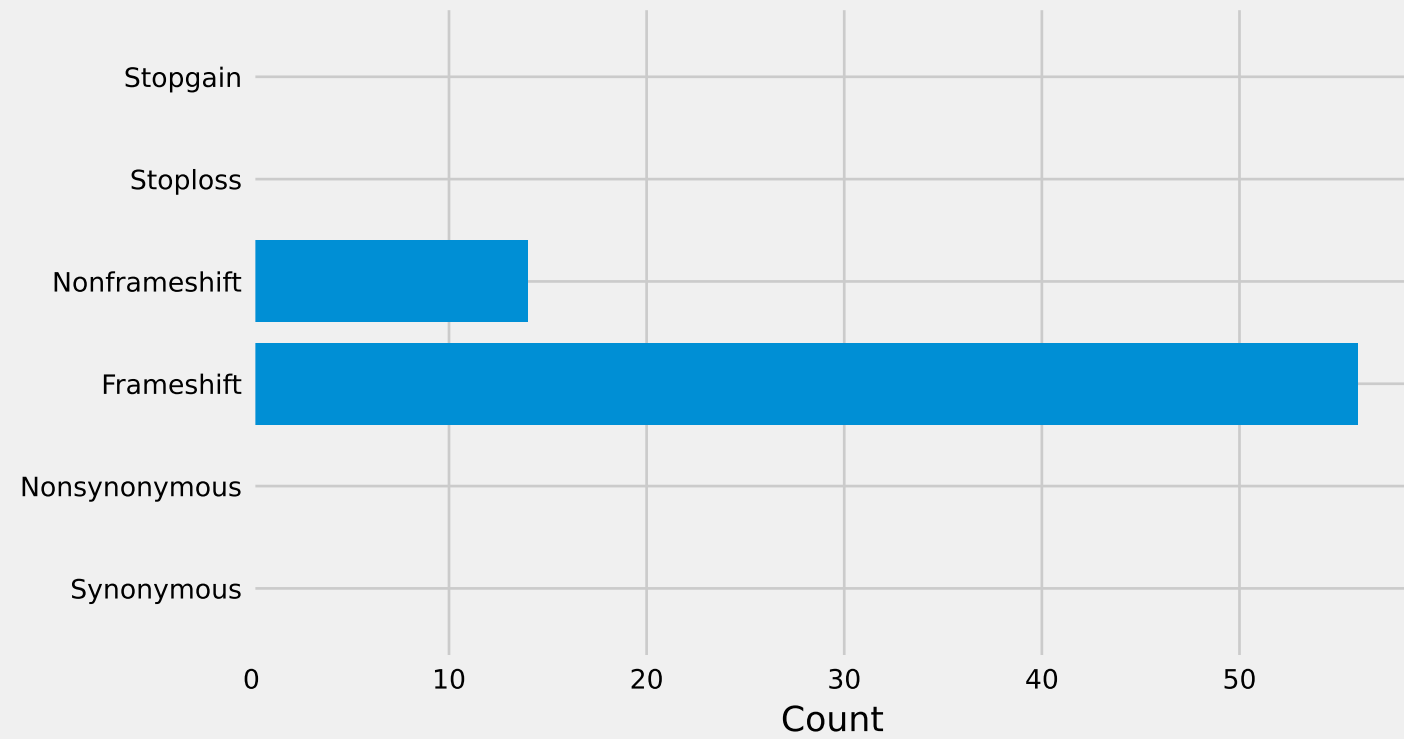

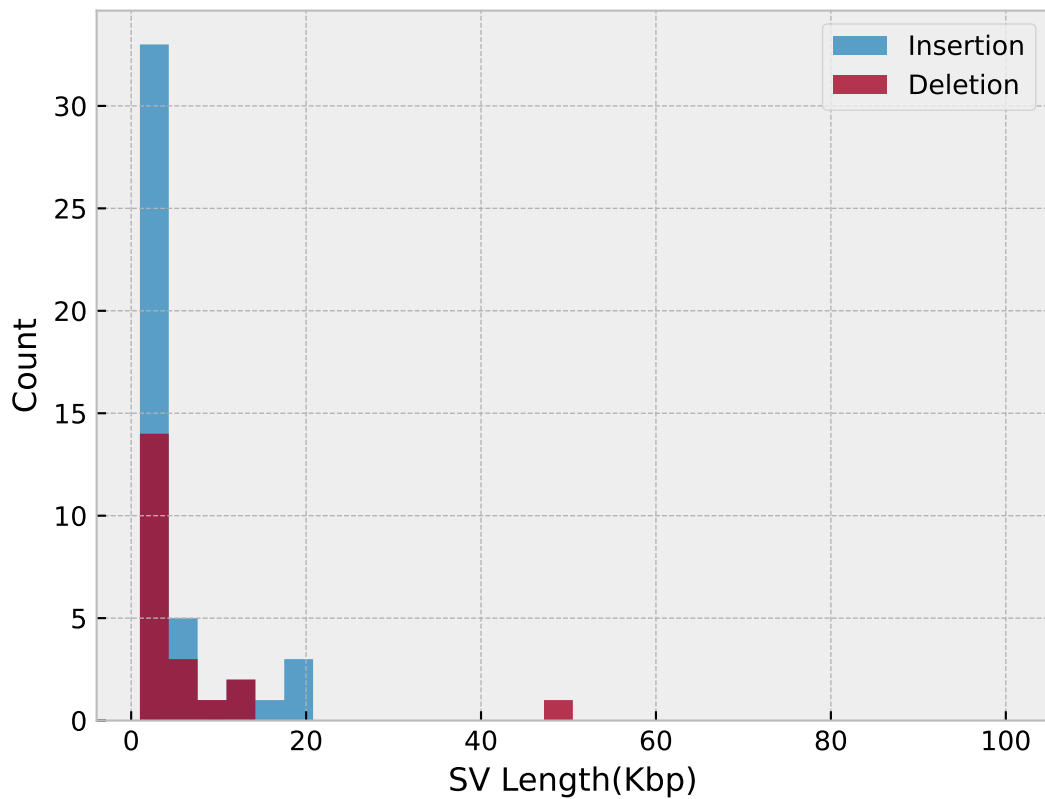

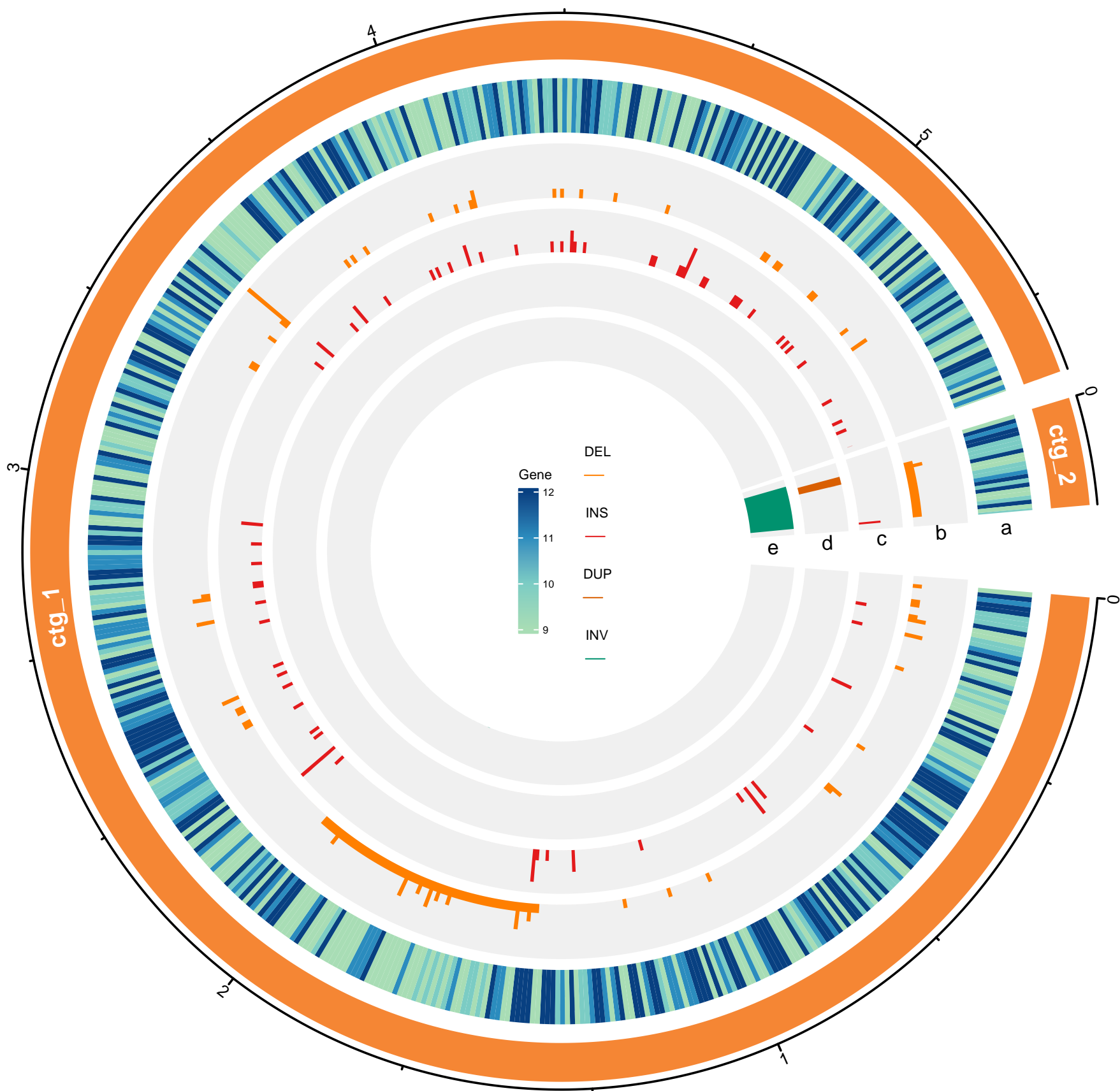

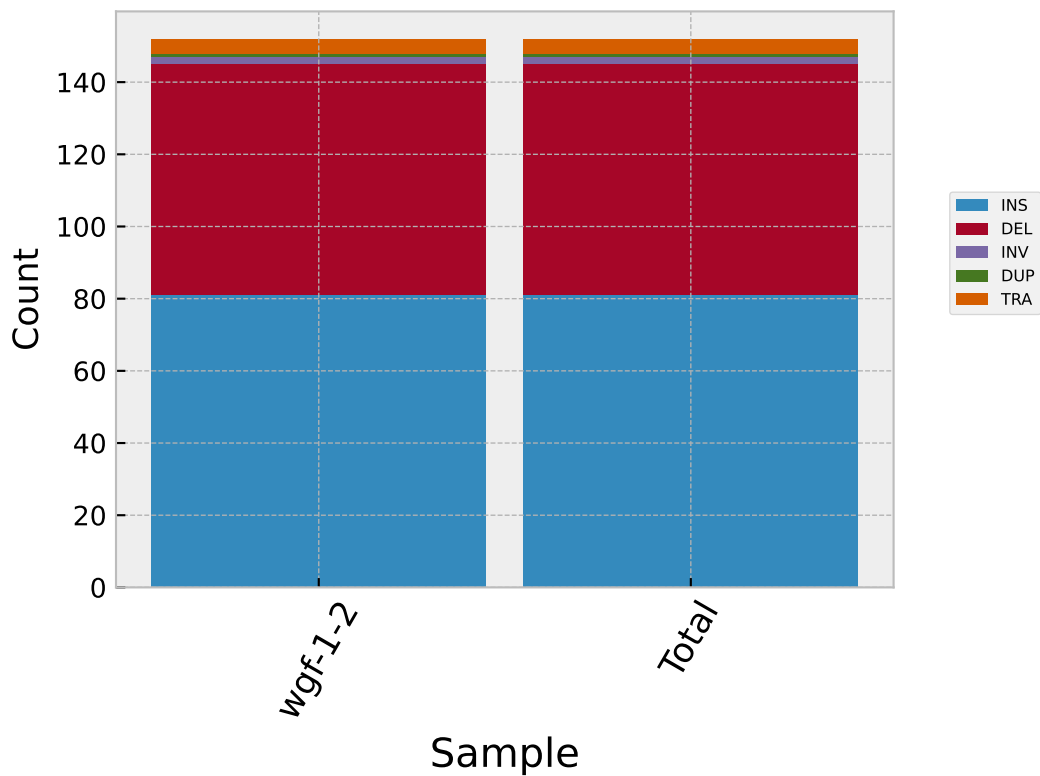

Supplement: Supplementary file 3 [file Image2.pdf]
