# Supplementary material for: Characterization of a Klebsiella pneumoniae mutant strain wGF 1–2 with attenuated virulence, altered morphology, and reduced biofilm formation
Source: Front Cell Infect Microbiol. 2026 Mar 27;16:1761564. doi: 10.3389/fcimb.2026.1761564 (PMC13066129; doi:10.3389/fcimb.2026.1761564)
Supplement: Supplementary file 2 [file Image1.pdf]

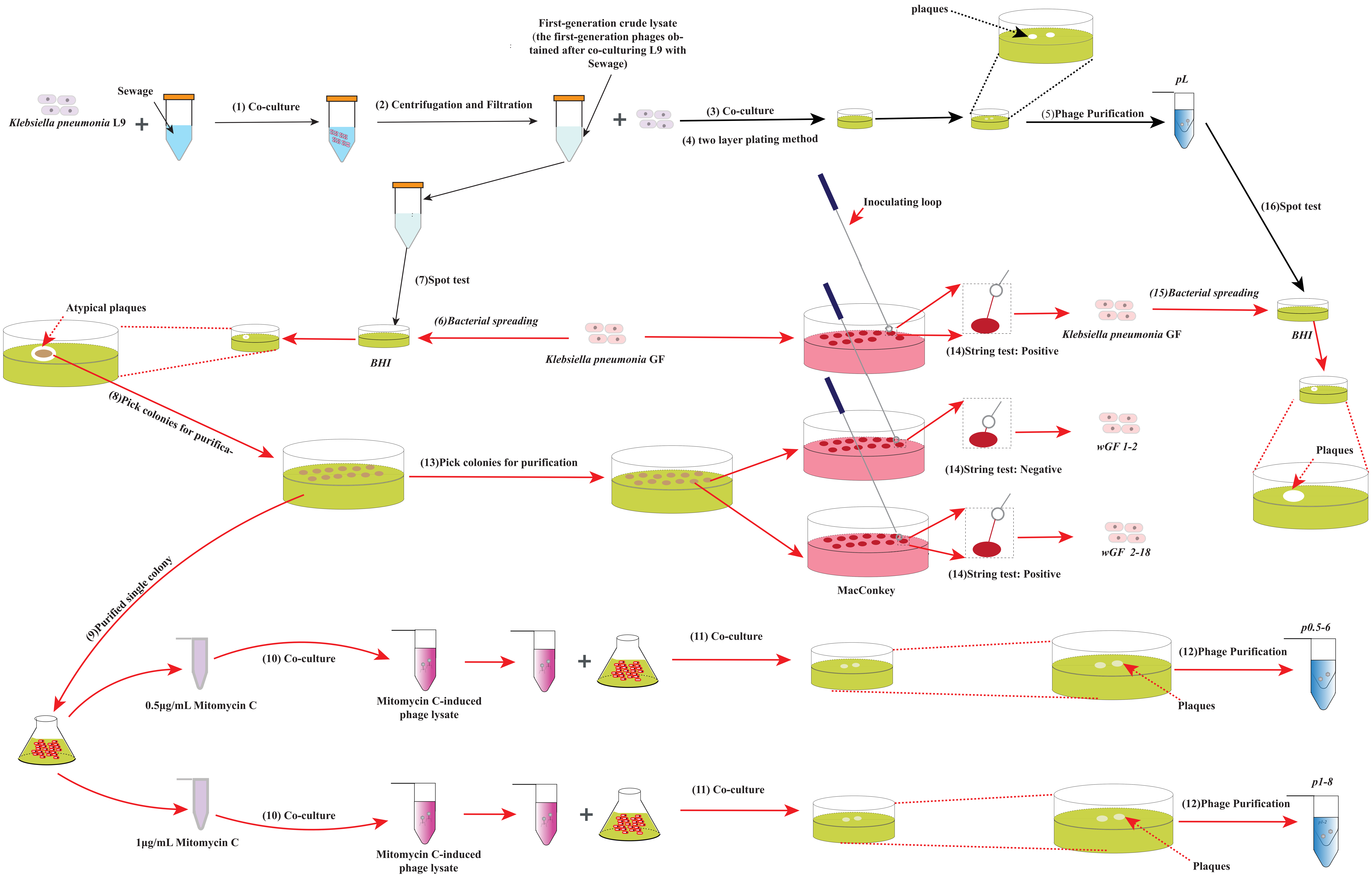

First-generation crude lysate (the first-generation phages obtained after co-culturing L9 with Sewage)
plaques
Sewage
Klebsiella pneumonia L9
(1) Co-culture
(2) Centrifugation and Filtration
(3) Co-culture
(4) two layer plating method
(5)Phage Purification
pL
(16)Spot test
Inoculating loop
(7)Spot test
Atypical plaques
(6)Bacterial spreading
BHI
Klebsiella pneumonia GF
(14)String test: Positive
Klebsiella pneumonia GF
(15)Bacterial spreading
BHI
(8)Pick colonies for purification
(13)Pick colonies for purification
wGF 1-2
(14)String test: Negative
Plaques
wGF 2-18
(14)String test: Positive
MacConkey
(9)Purified single colony
(11) Co-culture
p0.5-6
(12)Phage Purification
(10) Co-culture
0.5μg/mL Mitomycin C
Mitomycin C-induced phage lysate
Plaques
(11) Co-culture
p1-8
(12)Phage Purification
(10) Co-culture
1μg/mL Mitomycin C
Mitomycin C-induced phage lysate
Plaques

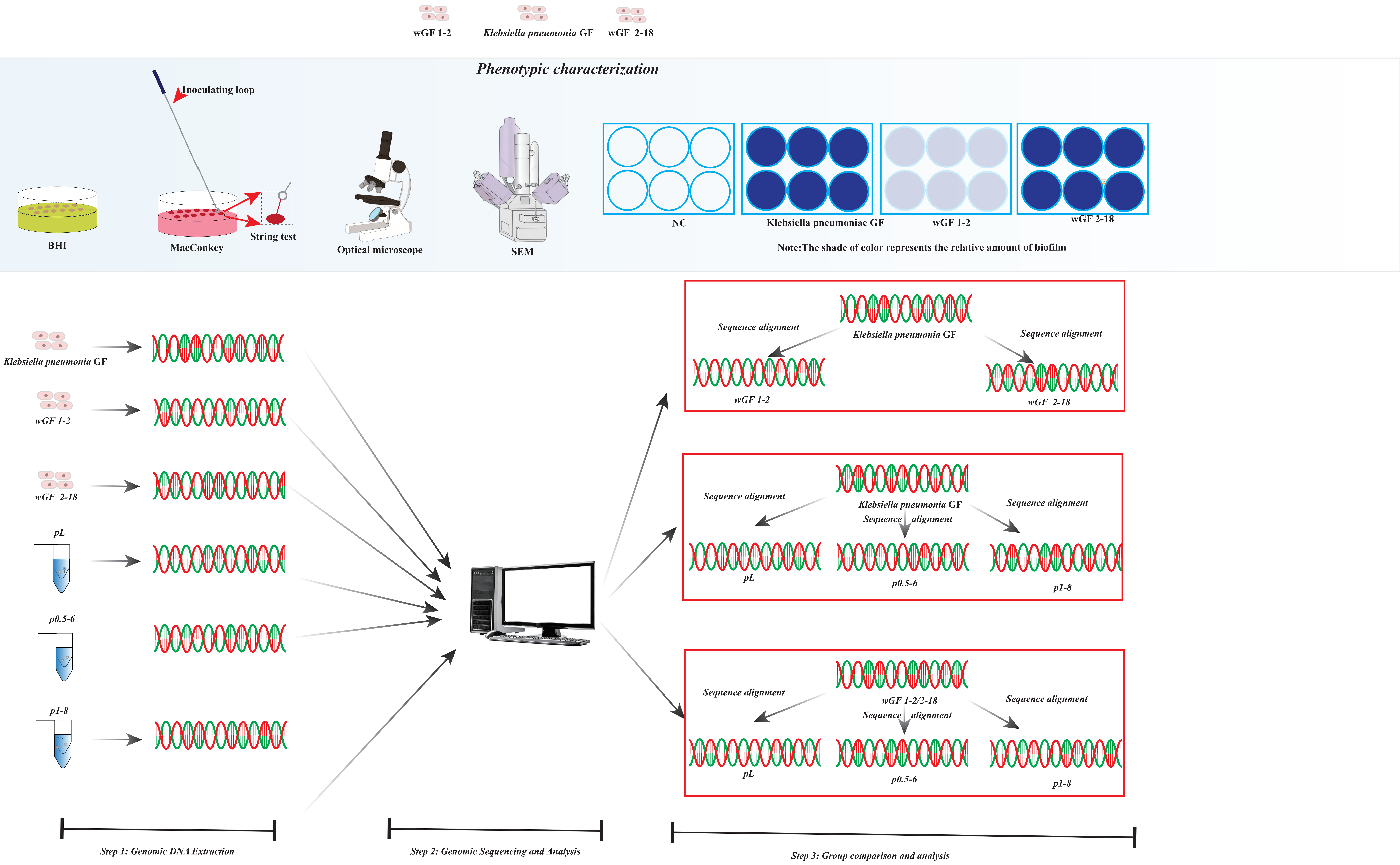

Note: Grouping instructions for Step 3
Group 1: GF VS wGF1-2/wGF 2-18 —— Using GF as the reference group, determine whether genomic structural variations exist between the two strains wGF1-2 and wGF 2-18;
Group 2: GF VS Three Phage Strains —— Exclude the possibility that the three phage strains are induced by endogenous prophages within GF;
Group 3: wGF1-2/wGF 2-18 GF VS Three Phage Strains —— Clarify whether the variant sequences in wGF1-2 and wGF 2-18 are contributed by the phages.

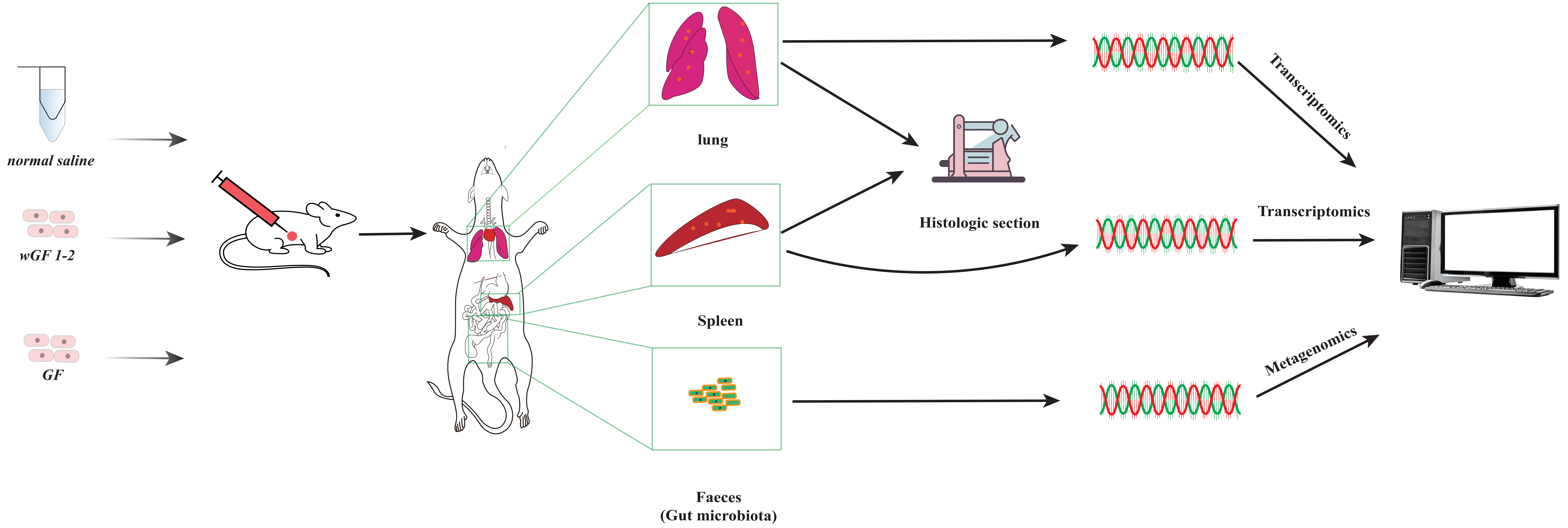
normal saline
wGF 1-2
GF
lung
Spleen
Faeces
(Gut microbiota)
Histologic section
Transcriptomics
Transcriptomics
Metagenomics
